# Author Correction: Posterior subthalamic nucleus (PSTh) mediates innate fear-associated hypothermia in mice

**DOI:** 10.1038/s41467-021-25434-5

**Published:** 2021-08-23

**Authors:** Can Liu, Chia-Ying Lee, Greg Asher, Liqin Cao, Yuka Terakoshi, Peng Cao, Reiko Kobayakawa, Ko Kobayakawa, Katsuyasu Sakurai, Qinghua Liu

**Affiliations:** 1 Peking University–Tsinghua University–NIBS Joint Graduate Program, School of Life Sciences, Tsinghua University, Beijing, China; 2 National Institute of Biological Sciences (NIBS), Beijing, China; 3 International Institute for Integrative Sleep Medicine (WPI-IIIS), University of Tsukuba, Tsukuba, Ibaraki Japan; 4 Tsinghua Institute of Multidisciplinary Biomedical Research (TIMBR), Tsinghua University, Beijing, China; 5 Department of Functional Neuroscience, Institute of Biomedical Science, Kansai Medical University, Osaka, Japan

**Keywords:** Emotion, Neural circuits

Correction to: *Nature Communications* 10.1038/s41467-021-22914-6, published online 11 May 2021.

The original version of this Article contained two errors in the Results section, in which the discussion of prior work published in reference 48 inaccurately referred to skin temperature, rather than tail temperature.

The sentence ‘It has recently been reported that optogenetic stimulation of axon terminals of PBel-CGRP+ (calcitonin gene-related peptide) neurons in PSTh induces a reduction in skin temperature^48^’ was inaccurate. The correct version replaces ‘skin temperature’ by ‘tail temperature’.

The sentence ‘These results are consistent with a recent report that opto-stimulation of the PBel-PSTh or PBel-CeA pathway can cause hypothermia^48^’ was incorrect, as the study in reference 48 studied tail temperature and not skin temperature and is thus not directly related to the findings on hypothermia reported in the original Article. This sentence has been removed.

This has been corrected in both the PDF and HTML versions of the Article.

