# Supplementary material for: Posterior subthalamic nucleus (PSTh) mediates innate fear-associated hypothermia in mice
Source: Nat Commun. 2021 May 11;12:2648. doi: 10.1038/s41467-021-22914-6 (PMC8113537; doi:10.1038/s41467-021-22914-6)
Supplement: Supplementary file 1 — Supplementary Information [file 41467_2021_22914_MOESM1_ESM.pdf]

Posterior subthalamic nucleus (PSTh) mediates innate fear-associated hypothermia in mice

Can Liu, Chia-Ying Lee, Greg Asher, Liqin Cao, Yuka Terakoshi, Peng Cao, Reiko Kobayakawa, Ko Kobayakawa, Katsuyasu Sakurai & Qinghua Liu

Supplementary Information

Supplementary Figure 1. *TRPA1* agonist cinnamaldehyde treatment and 2MT induced c-fos expression.

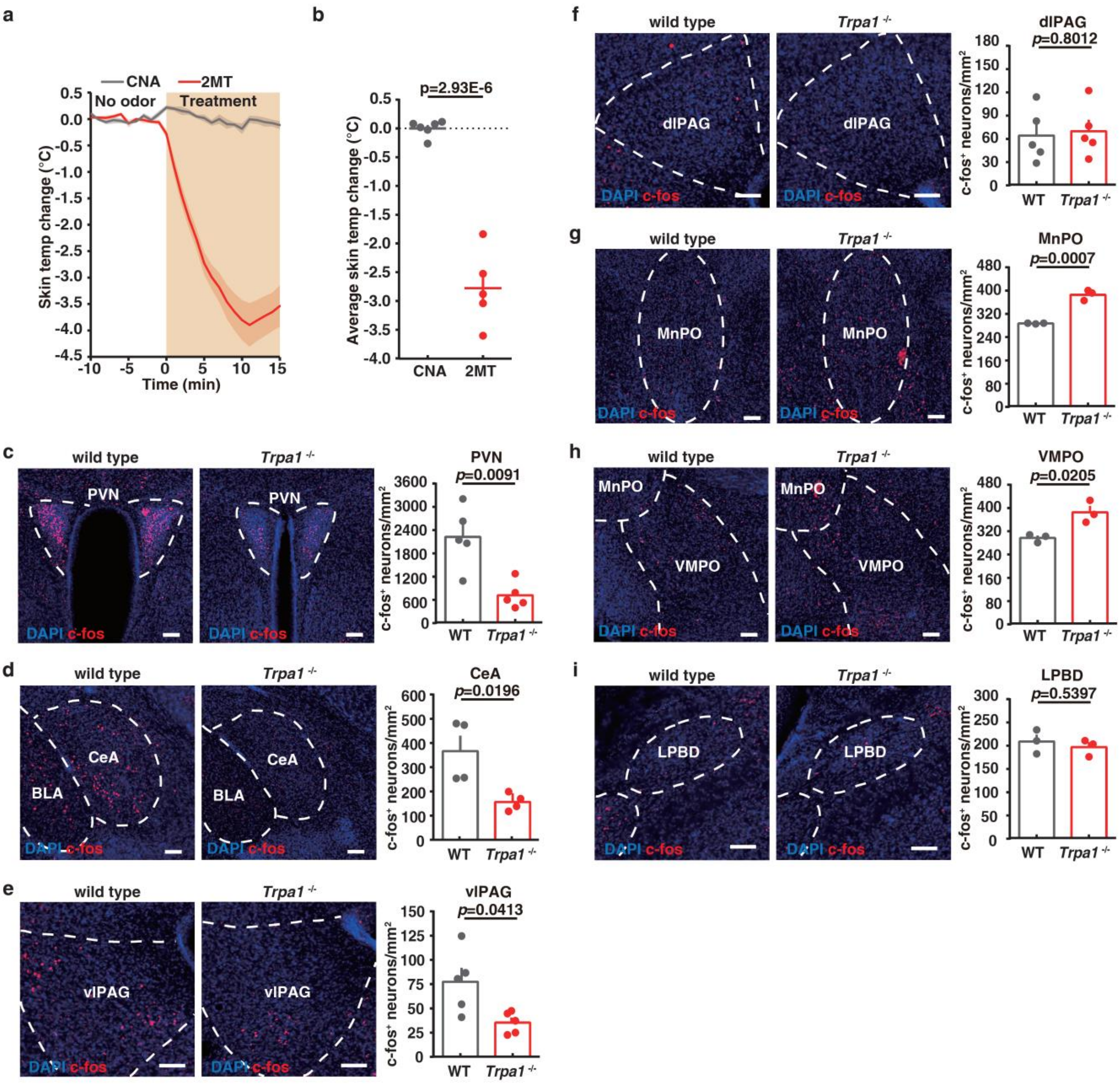

Supplementary Figure 1. TRPA1 agonist cinnamaldehyde treatment and 2MT induced c-fos expression.

a Skin temperature curves of wild-type mice before and during 2MT (n=5) or cinnamaldehyde (n=6) treatment. **b** Average skin temperature changes of wild-type during 2MT or cinnamaldehyde treatment. **c-I** Representative images and quantitative analysis of 2MT-evoked c-fos expression showing 2MT-induced c-fos expression in PVN (**c**), CeA (**d**), vlPAG (**e**), dlPAG (**f**), MnPO (**g**), VMPO (**h**), LPBD (**i**) of wild-type and *Trpa1*$^{-/-}$ mice (**c**, **e**, **f**, n=5; **d**, n=4; **g**, **h**, **I**, n=5). Data are mean ± SEM; two-side Student's t-test. Scale bars, 100 µm. CNA, cinnamaldehyde; MnPO, median preoptic nucleus; VMPO, ventromedial preoptic nucleus; PVN, paraventricular hypothalamic nucleus; CeA, central amygdala; vlPAG, ventrolateral periaqueductal gray; dlPAG, dorsolateral periaqueductal gray; LPBD, dorsal part of the lateral parabrachial nucleus.

Supplementary Figure 2. Retrograde tracing from NTS with CTB.

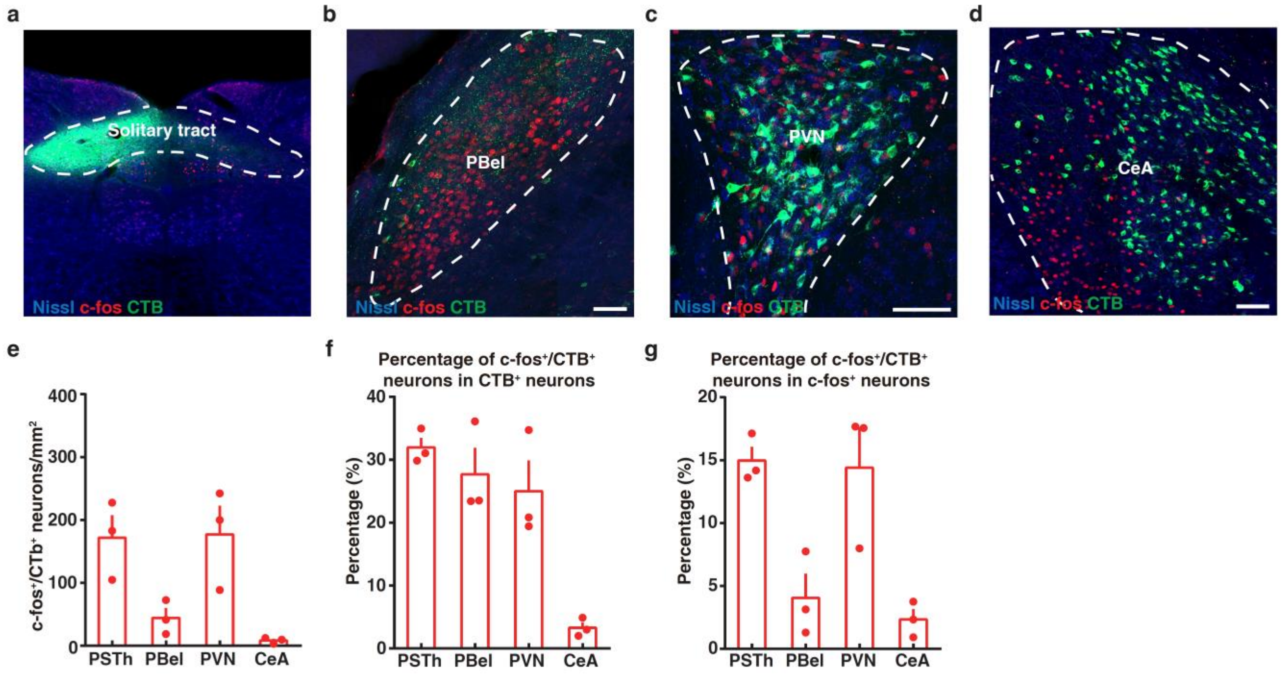

Supplementray Figure 2. Retrograde tracing from NTS with CTB.

a Representative image showing CTB injection site in NTS (n=3). **b-d** Representative images showing double immunostaining of CTB and c-fos in PBel (**b**), PVN (**c**), and CeA (**d**) (PSTh is shown in Fig. 2b) (n=3). **e** Quantitative analysis of the density of c-fos$^+$, CTB$^+$ double-positive neurons in various brain regions (n=3). **f** Quantitative analysis of the percentage of CTB$^+$ neurons that are also c-fos$^+$ in various brain regions (n=3). **g** Quantitative analysis of the percentage of c-fos$^+$ neurons that are also CTB$^+$ in various brain regions (n=3). **(e-g)** Data are mean ± SEM. Scale bars, 100 µm.

Supplementary Figure 3. Inhibitory effect of TeLC depends on the virus transduction rate.

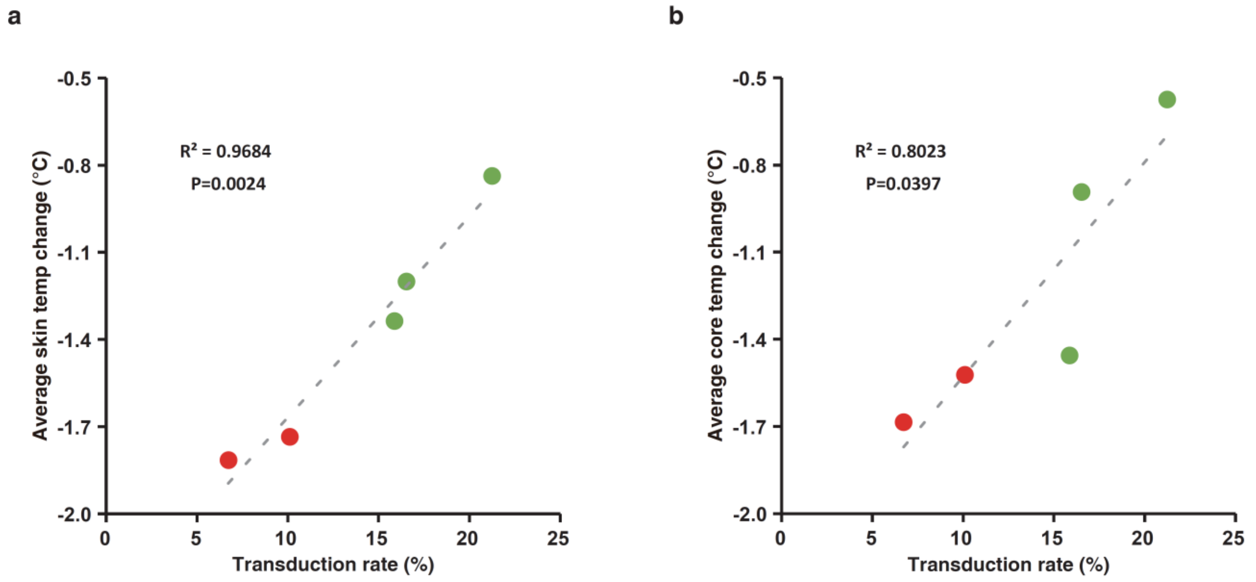

Supplementary Figure 3. Inhibitory effect of TeLC depends on the virus transduction rate.

a Quantitative analysis showing the 2MT-evoked average skin temperature reduction of TeLC-expressing mice is inversely related to the virus transduction rate. **b** Quantitative analysis showing the 2MT-evoked average core body temperature reduction of TeLC-expressing mice is inversely related to the virus transduction rate. Linear regression was used for statistical analysis. The two red dots in each graph refers to two mice with low virus transduction rate, which were not included in Figure 3.

Supplementary Figure 4. Fiber implant sites of ChR2 labled mice in NTS.

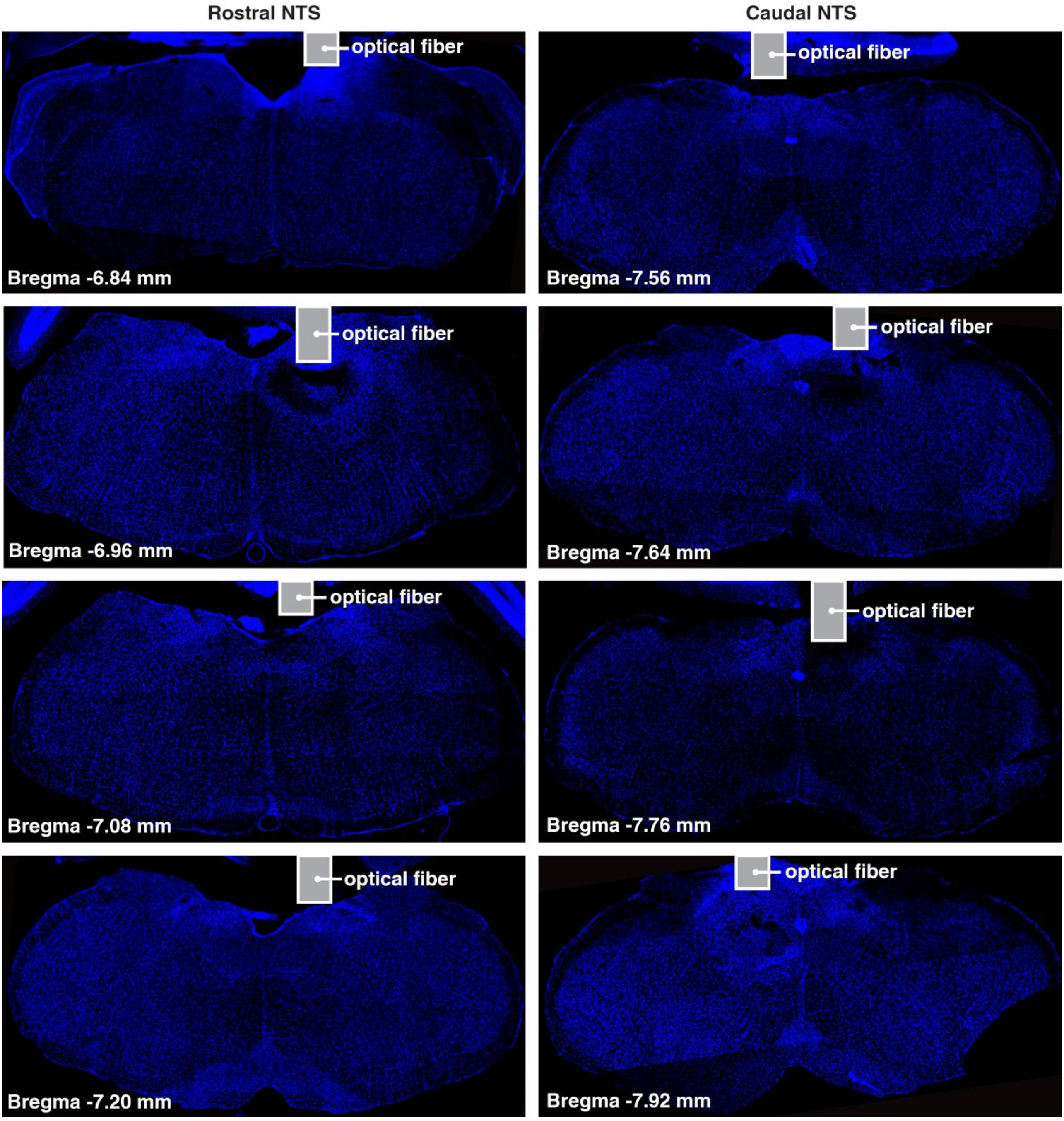

Supplementary Figure 4. Fiber implant sites of ChR2 labeled mice in NTS.

Images showing optical fiber implant site in NTS.

Supplementary Figure 5. Retrograde tracing from PSTh with CTB.

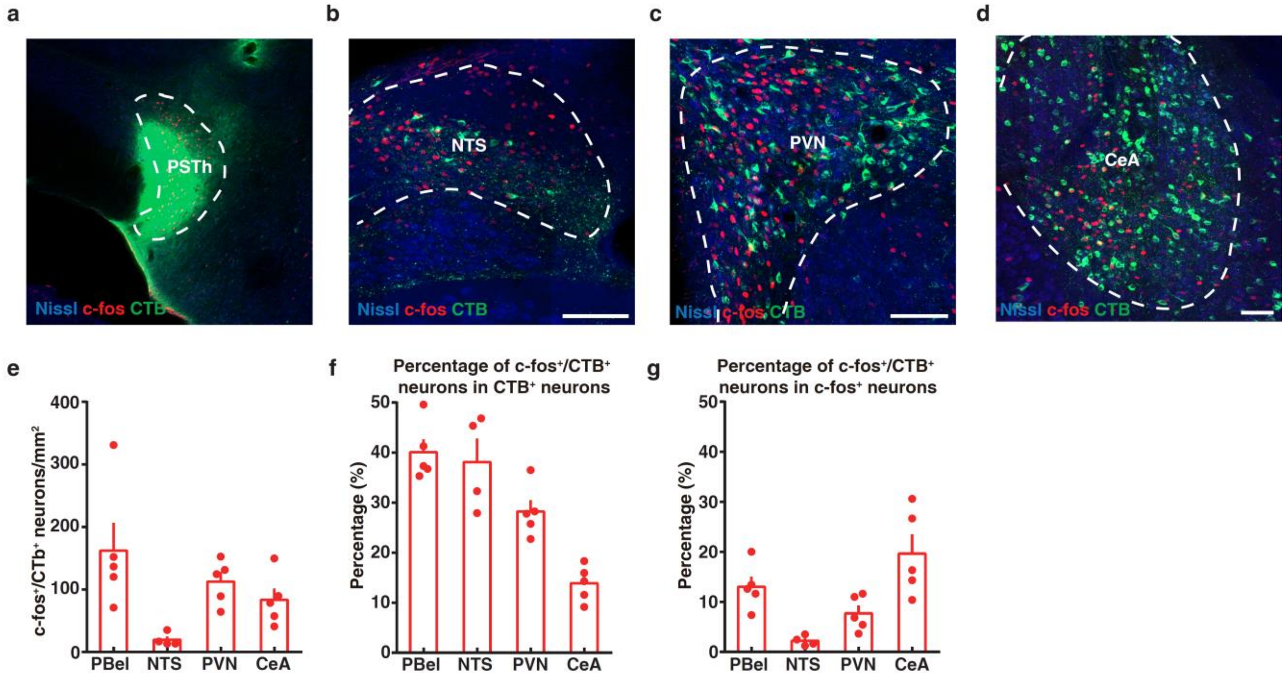

Supplementary Figure 5. Retrograde tracing from PSTh with CTB.

a Representative image showing CTB injection site in PSTh. **b-d** Representative images showing double immunostaining of CTB and c-fos in NTS (**b**), PVN (**c**), and CeA (**d**) (PBel is shown in Fig. 5b) (n=5). **e** Quantitative analysis of the density of c-fos$^+$, CTB$^+$ double-positive neurons in various brain regions (n=5). **f** Quantitative analysis showing the percentage of CTB$^+$ positive neurons that are also c-fos$^+$ in various brain regions (n=5). **g** Quantitative analysis of the c-fos$^+$ neurons that are also CTB$^+$ in various brain regions (n=5). **(e-g)** Data are mean ± SEM. Scale bars, 100 µm.

Supplementary Figure 6. Opto-stimulation of PBel-CeA pathway evokes mild hypothermia.

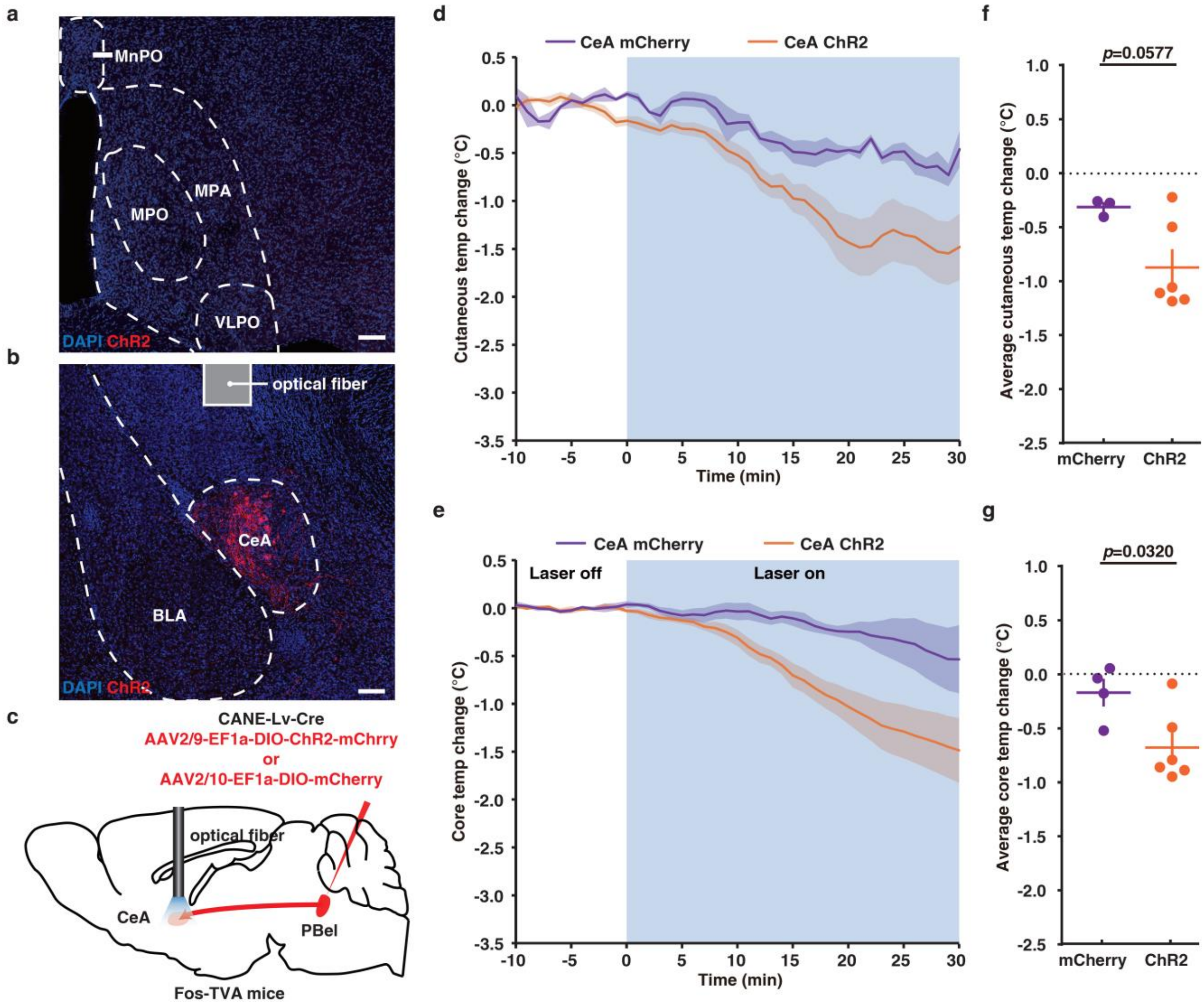

Supplementary Figure 6 Opto-stimulation of PBel-CeA pathway evokes mild hypothermia.

a, b Representative images showing that CANE-labeled PBel neurons project to CeA (b), but not POA (a) in Fos^{TVA} mice (n=2). **c** Schematic of selective opto-stimulation of the axon terminals in CeA from 2MT-activated PBel neurons labeled by CANE in Fos^{TVA} mice. **d, e** Skin (**d**) and core (**e**) temperature curves of mCherry-expressing (n=3 for skin, n=4 for core) and ChR2-expressing (n=6) Fos^{TVA} mice before and during photoactivation of the axon terminals of 2MT-activated PBel neurons in CeA. **f, g** Average skin (**f**) and core (**g**) temperature changes of mCherry-expressing and ChR2-expressing Fos^{TVA} mice during photoactivation of the axon terminals of 2MT-activated PBel neurons in CeA. **(d-g)** Data are mean ± SEM; two-side Student's t-test. Scale bars, 100 µm.

Supplementary Figure 7. Inhibition of vGlut2 ⁺ PBel neurons attenuates 2MT-evoked hypothermia.

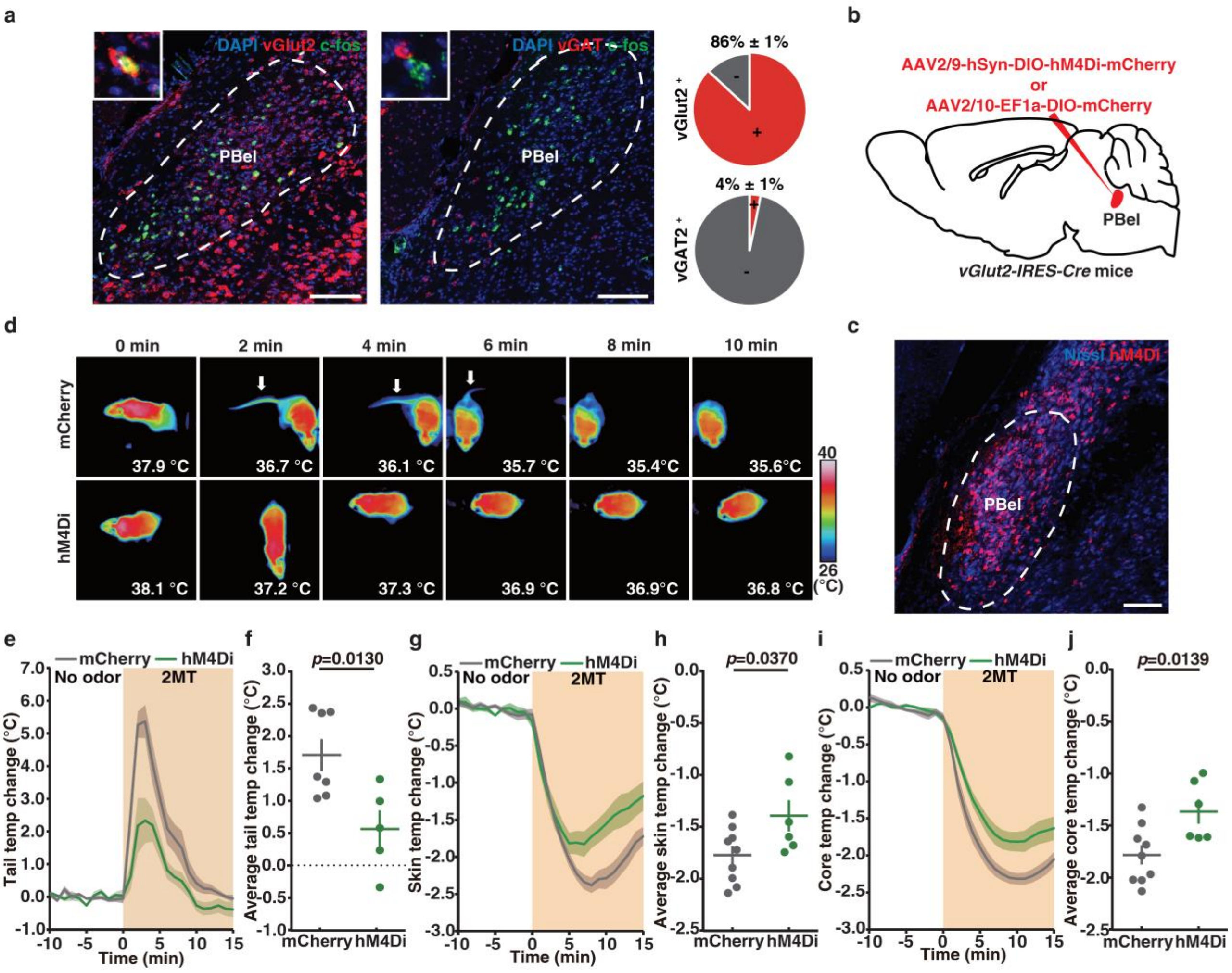

Supplementary Figure 7 Inhibition of vGlut2$^+$ PBel neurons attenuates 2MT-evoked hypothermia and tail temperature increase.

a Representative images and quantitative analysis showing the percentage of *c-fos*$^+$*, vGlut2*$^+$ and *c-fos*$^+$*, vGAT*$^+$ double-positive neurons among 2MT-activated *c-fos*$^+$ neurons in PBel by two-color in situ hybridization (n=3). **b** Schematic of chemogenetic inhibition of vGlut2$^+$ PBel neurons in *vGlut2-IRES-Cre* mice. **c** Representative image showing hM4Di-labeled vGlut2$^+$ PBel neurons (n=4). **d** Time-lapsed thermal images of mCherry-expressing and hM4Di-expressing mice during 2MT treatment following administration of C21. **e, g, i** Tail (**e**), skin (**g**) and core (**i**) temperature curves of mice with (hM4Di, n=6) and without (mCherry, n=9) inactivation of vGlut2$^+$ PBel neurons before and during 2MT treatment. **f, h, j** Average tail (**f**), skin (**h**) and core (**j**) temperature changes of mice with (hM4Di) or without (mCherry) inactivation of vGlut2$^+$ PBel neurons during 2MT treatment. A few mice were not included in the analysis of tail temperature (**e, f**) because their tails were frequently obscured in the thermal images. **(e-j)** Data are mean ± SEM; two-side Student's t-test. Scale bars, 100 µm.

Supplementary Table Primers used to produce probes of in situ hybridization

Probe	Primer	Sequence
c-fos	c-fos-F	AGCAGTGACCGCGCTCCCACCCAGC
	c-fos-R_T7	CGCGCGTAATACGACTCACTATAGGGCAGACCACCTCGACAATGCATGATCAG
vGlut2	vGlut2-F	CCAAATCTTACGGTGCTACCTC
	vGlut2-R_T7	CGCGCGTAATACGACTCACTATAGGGTAGCCATCTTTCCTGTTCCACT
vGAT	vGAT-F	GCCATTCAGGGCATGTTC
	vGAT-R_T7	CGCGCGTAATACGACTCACTATAGGGAGCAGCGTGAAGACCACC
