# Supplementary material for: Posterior subthalamic nucleus (PSTh) mediates innate fear-associated hypothermia in mice
Source: Nat Commun. 2021 May 11;12:2648. doi: 10.1038/s41467-021-22914-6 (PMC8113537; doi:10.1038/s41467-021-22914-6)
Supplement: Supplementary file 3 — Reporting Summary [file 41467_2021_22914_MOESM3_ESM.pdf]

nature research

Corresponding author(s): Qinghua Liu

Last updated by author(s): 2021/3/5

Reporting Summary

Nature Research wishes to improve the reproducibility of the work that we publish. This form provides structure for consistency and transparency in reporting. For further information on Nature Research policies, see our Editorial Policies and the Editorial Policy Checklist.

Statistics

For all statistical analyses, confirm that the following items are present in the figure legend, table legend, main text, or Methods section.

n/a	Confirmed	
☐	☒	The exact sample size (*n*) for each experimental group/condition, given as a discrete number and unit of measurement
☐	☒	A statement on whether measurements were taken from distinct samples or whether the same sample was measured repeatedly
☐	☒	The statistical test(s) used AND whether they are one- or two-sided<br>*Only common tests should be described solely by name; describe more complex techniques in the Methods section.*
☒	☐	A description of all covariates tested
☐	☒	A description of any assumptions or corrections, such as tests of normality and adjustment for multiple comparisons
☐	☒	A full description of the statistical parameters including central tendency (e.g. means) or other basic estimates (e.g. regression coefficient) AND variation (e.g. standard deviation) or associated estimates of uncertainty (e.g. confidence intervals)
☐	☒	For null hypothesis testing, the test statistic (e.g. *F*, *t*, *r*) with confidence intervals, effect sizes, degrees of freedom and *P* value noted<br>*Give P values as exact values whenever suitable.*
☒	☐	For Bayesian analysis, information on the choice of priors and Markov chain Monte Carlo settings
☒	☐	For hierarchical and complex designs, identification of the appropriate level for tests and full reporting of outcomes
☒	☐	Estimates of effect sizes (e.g. Cohen's *d*, Pearson's *r*), indicating how they were calculated

Our web collection on statistics for biologists contains articles on many of the points above.

Software and code

Policy information about availability of computer code

Data collection	Zeiss software Zen, infrared digital thermographic camera (H2640, NIPPON AVIONICS), telemetry transmitter (TA11TA-F10, DSI)
Data analysis	Microsoft Excel, GraphPad Prism6, Photoshop CS6, Image J

For manuscripts utilizing custom algorithms or software that are central to the research but not yet described in published literature, software must be made available to editors and reviewers. We strongly encourage code deposition in a community repository (e.g. GitHub). See the Nature Research guidelines for submitting code & software for further information.

Data

Policy information about availability of data

All manuscripts must include a data availability statement. This statement should provide the following information, where applicable:

- Accession codes, unique identifiers, or web links for publicly available datasets
- A list of figures that have associated raw data
- A description of any restrictions on data availability

All raw-data is available upon request.

Field-specific reporting

Life sciences study design

All studies must disclose on these points even when the disclosure is negative.

Sample size	We chose at least three independent biological replicates, which are standard numbers for the field, to meet the statistical requires. No method was used to calculate the sample size.
Data exclusions	Data was not exclude from experiments unless apparent failures.
Replication	Temperature recording results were consistently replicated across at least three individual mice in each group. Immunostaining and two-color in situ hybridization were replicated at least twice. All replicates generating similar results. Details for each experiment were described in the text and figure legends.
Randomization	All experiments were randomly assigned.
Blinding	The investigators were not blinded to the design of the study. Blinding during temperature recording and data analysis was not needed because conditions were well controlled and data was collected and analyzed by computer automatically. Blinding is not necessary for cell counting analysis because the results are quantitative and did not require subjective judgment or interpretation.

Reporting for specific materials, systems and methods

We require information from authors about some types of materials, experimental systems and methods used in many studies. Here, indicate whether each material, system or method listed is relevant to your study. If you are not sure if a list item applies to your research, read the appropriate section before selecting a response.

Materials & experimental systems

n/a	Involved in the study
☐	☒ Antibodies
☒	☐ Eukaryotic cell lines
☒	☐ Palaeontology and archaeology
☐	☒ Animals and other organisms
☒	☐ Human research participants
☒	☐ Clinical data
☒	☐ Dual use research of concern

Methods

n/a	Involved in the study
☒	☐ ChIP-seq
☒	☐ Flow cytometry
☒	☐ MRI-based neuroimaging

Antibodies

Antibodies used	1st antibodies: Rabbit polyclonal anti-c-fos (Sigma-Aldrich, ABE457), Goat polyclonal anti-mCherry (SCIGEN, AB0040-200), Rat monoclonal anti-GFP (Nacalai tesque, 04404-84); All 2nd antibodies were obtained from Jackson ImmunoResearch: Donkey anti-rabbit Alexa Fluor 488 (711-545-152), Donkey anti-rabbit Cy3 (711-165-152), Donkey anti-rat Alexa Fluor 488(712-545-153), Donkey anti-goat Cy3 (705-165-147).
Validation	Rabbit polyclonal anti-c-fos (Sigma-Aldrich, ABE457): https://www.sigmaaldrich.com/catalog/product/mm/abe457?lang=ja&region=JP<br>Goat polyclonal anti-mCherry: http://www.sicgen.pt/product/mcherry-polyclonal-antibody_1_27<br>Rat monoclonal anti-GFP: https://www.nacalaiusa.com/products/view/101/anti-gfp-rat-igg2a-monoclonal-gf090r<br>All 2nd antibodies : https://www.jacksonimmuno.com

Animals and other organisms

Policy information about studies involving animals; ARRIVE guidelines recommended for reporting animal research

Laboratory animals	C57BL/6J (CLEA, Japan), Trpa1-/- (stock 006401), FosTVA (stock 027831), vGlut2-IRES-Cre (stock 016963). Male mice of more than 8 weeks of age were used for experiments.
Wild animals	No wild animal used in this study.
Field-collected samples	No samples collected at the filed in this study.
Ethics oversight	Animal protocols used in this study were approved by the Institutional Animal Care and Use Committee of International Institute for Integrative Sleep Medicine at University of Tsukuba, Japan and the National Institute of Biological Sciences, Beijing, China.

Note that full information on the approval of the study protocol must also be provided in the manuscript.
